# Comparative Evaluation of Locally Applied Chlorhexidine 1% Gel and *Citrus sinensis* Peel Extract Gel as an Adjunct to Nonsurgical Periodontal Therapy in Localized Periodontitis Treatment: A Clinico‐Microbiological Study

**DOI:** 10.1155/ijod/2038398

**Published:** 2025-12-28

**Authors:** Ishika Sarkar, Pritish Chandra Pal, Swet Nisha, Debika Karmakar, S. R. Savan, Dhanraj Budhai

**Affiliations:** ^1^ Department of Periodontology, Haldia Institute of Dental Sciences and Research, Haldia, West Bengal, India, icare-haldia.org; ^2^ Department of Periodontology, University of Guyana School of Dentistry, Georgetown, Guyana

**Keywords:** antimicrobial, antioxidant, chlorhexidine, *Citrus sinensis*, controlled release drugs, local drug delivery, periodontitis

## Abstract

**Background:**

Periodontal disease is polymicrobial in nature and to control the infection we need to target the inflammatory component. Nonsurgical periodontal therapy is used for mechanical debridement. Use of agents along with nonsurgical periodontal therapy is gaining momentum. Locally delivered agents like antibiotics and herbal products are used in nonsurgical and surgical periodontal therapy. Phytotherapy offers antioxidant, antimicrobial action and has less chances of drug resistance.

**Objective:**

This study design compared locally delivered chlorhexidine (CHX) 1% gel and *Citrus sinensis* peel extract (CSPE) gel in the management localized periodontitis as an adjuvant treatment to scaling and root planing (SRP).

**Material and Method:**

Forty‐six subjects diagnosed with Grade II/III localized periodontitis were randomly assigned into two groups. After thorough SRP, CSPE gel placement was done in the test group and 1% CHX gel in the control group. Clinical parameters measured were probing pocket depth (PPD), clinical attachment level (CAL) and microbiological analysis (bacterial culture from pocket sample) was done prior to SRP and at 60 days.

**Results:**

Both the 1% CHX and CSPE gel provide comparable outcome (*p* ≥ 0.05) in improvement of PPD, CAL and bacterial colony count. Statistically significant results were obtained in terms of PPD; *p* = 0.044 (test group) and *p* = 0037 (control group). CAL showed statistically significant results in test group with *p* = 0.044. Inter group and Intra group comparison of plaque index showed no statistical difference. Colony forming units (CFU) between the two groups (*p* = 0.001) was found statistically significant.

**Conclusion:**

Locally delivered herbal CSPE gel can be an effective, economical,and antibacterial gel as an adjunctive treatment of periodontitis.

## 1. Introduction

Chronicperiodontitis is an inflammatory disease which is caused by accumulation of biofilm which subsequently leads to disintegration of periodontal tissues followed by tooth loss. To address the infection, we need to resolve the inflammation [1]. The oxidative and antioxidant balance is disrupted which leads to microbial proliferation and dysbiosis. The journey from dysbiosis to symbiosis needs alteration at immune level with various cell interactions both at macro and micro levels [2]. Nonsurgical periodontal therapy helps in mechanical removal of biofilm and creates favorable environment for restoration of periodontal health. Scaling and root planing (SRP) involves deep mechanical removal of the infectious biofilm attached to the tooth surface. Recently, adjuvant use of local drug delivery agents along with nonsurgical periodontal therapy has been proven advantageous in terms of early healing and maintenance of periodontal health [3]. Local drug delivery agents have antimicrobial action and act locally. They are available in fibers, gels, nanoparticles for ease of placement [3]. Tetracycline fibers, metronidazole, chlorhexidine (CHX) chips are commonly used local drug agents. Herbal drugs like aloe vera, pomegranate,and curcumin have been used as local drug delivery agents [4]. Phytotherapy is the term coined for use of plant‐based natural agent for promoting health.

In periodontal therapy various phytotherapy agents have been used with minimal side effects. Orange, a tasty, juicy, citrus fruit of Rutaceae family is scientifically known as *Citrus sinensis*.The extract of this fruit peel *Citrus sinensis* peel extract (CSPE) is of medicinal value as it contains flavonoids, vitamin C, tannins, and saponins [5]. Various types of flavonoids namely hesperidin, rutin, naringin, eriocitrin, and quercetin exist in orange peel, hesperidin being the most common [6]. Hesperidin mostly found in *Citrus sinensis* compared to other oranges has antioxidant, anti‐inflammatory, antiadipogenic, antiallergic, antiviral, and anticarcinogenic properties in addition to an angiogenic element that may promote the development of new blood vessels [7]. Additionally, hesperidin acts as an immunomodulator, stimulates release of growth factors, such as fibronectin, fibroblast stimulating factor, and collagenase which stimulate the proliferation of fibroblasts [8]. The modus operandi of drug therapy involves the availability of the antimicrobial agent at the pathological site in sufficient concentration andfor adequate duration to kill and obstruct growth of subgingival microorganisms [9]. Higher concentration at subgingival sites offers a natural pool with gingival crevicular fluid (GCF), and enables easy distribution of the drug throughout the pocket [10]. Hence, periodontal pocket forms the most favorable site for placement of LDDs.

CHX is an antimicrobial agent having both systemic and local effects in periodontal disease management. CHX, due to its bactericidal as well as bacteriostatic properties and has broad spectrum of action, thereby justifying its application as an effective topical agent that can significantly reduce the number of oral microorganisms in a short duration [11]. Ciriminna et al. [12] demonstrated the antimicrobial, antibiofilm, anti‐inflammatory, antioxidant, and anticaries effect of *Citrus sinensis*. However, in our current knowledge there are very few studies have been published using orange peel extract gel as LDD in periodontitis patients, hence it is justified to use orange extract gel as LDD in periodontitis patients. In the present study, we compared locally delivered CHX 1% gel and CSPE gel as an adjuvant to Phase I therapy in localized periodontitis treatment.

## 2. Material and Methods

This clinico‐microbiological study was executed in the Department of Periodontics at a tertiary hospital. The study period was from May 2024 to October 2024 with total study duration of 60 days. Total 46 subjects diagnosed with Grade II/III localized periodontitis were randomly allocated into two groups by using coin toss method. Control group received SRP + CHX1% gel as LDD while test group received SRP + CSPE gel as LDD. Ethical approval was taken from institutional ethical committee (HIDSAR/ETHICS/324/2024). Written consent was obtained from the study participants.

Inclusion criteria included subjects with age between 18 and 60 years, systemically healthy subjects, patients having at least 20 teeth present and suffering from Grade II/III periodontitis with probing pocket depth (PPD) ≥ 4 mm and ≤ 6 mm and who have signed the consent form. Subjects with good oral hygiene post Phase I therapy completion were included in this study.

Exclusion criteria included subjects with history of systemic diseases, history of chemotherapy or radiotherapy, subjects requiring prophylactic antibiotics, heavy smokers, pregnancy or lactation, and patients with poor oral hygiene.

### 2.1. Preparation of the *Citrus sinensis* Peel Extract

**Figure 1:**
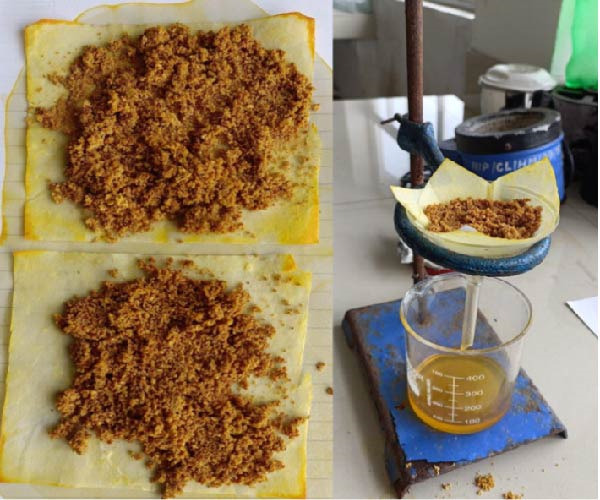
Filtration of *citrus sinensis* peel soaked in petroleum ether.

**Figure 2:**
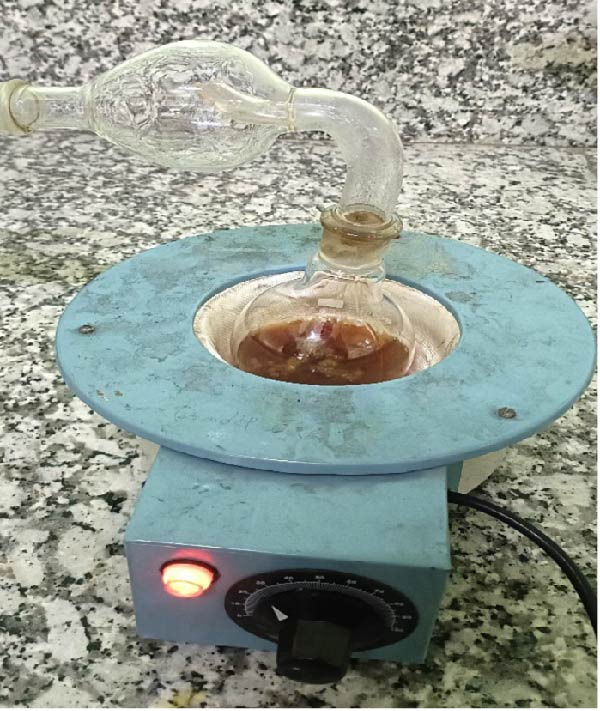
Methanol evaporation by distillation to collect liquid extract.

Orange peels were separated from the fruit after purchasing from local market. Careful washing of peel followed by air drying was done at room temperature (30°C) for 2 to 3 days. This was followed by pulverization to a fine powder and stored in airtight bottles. For extract preparation, 100 gm powder was dissolved in 250 mL petroleum ether in an airtight container and kept aside for 48 h with occasional shaking. Whatman No. 1 filter paper was used to filter the solution after 48 h and the lipophilic filtrate was collected (Figure 1). Hot air oven was used to dry the orange peel residue and the dried powder was then soaked in 250 mL methanol in air tight container and kept aside for 72 h. This was followed by filtration using a cotton cloth and the liquid extract collected. Methanol was then evaporated from the liquid by the process of distillation and the pure orange peel extract was obtained (Figure 2) [13].

### 2.2. Preparation of the *Citrus sinensis* Gel

**Figure 3:**
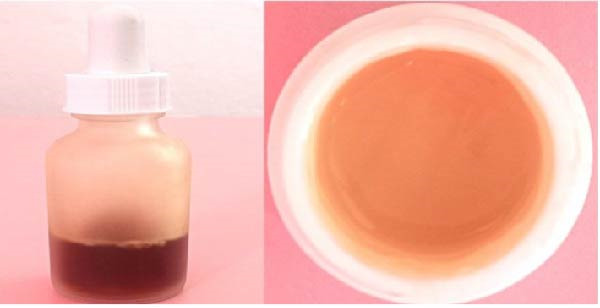
CSPE extract and gel.

Carbopol 940 (1 g) was dissolved in a beaker with 30 mL of distilled water solution and clear gel was prepared using a magnetic stirrer for 30 min with intermittent stirring. Further, methyl paraben 0.5 gm was mixed in distilled water (5 mL). Further propylene glycol 400 was added once the solution settles at room temperature. *Citrus sinensis* extract was combined to the above mixture. Continuous stirring with drops of triethanolamine resulted in gel formation with pH 6.8–7. (Figure 3) [14].

### 2.3. Periodontal Treatment

**Figure 4:**
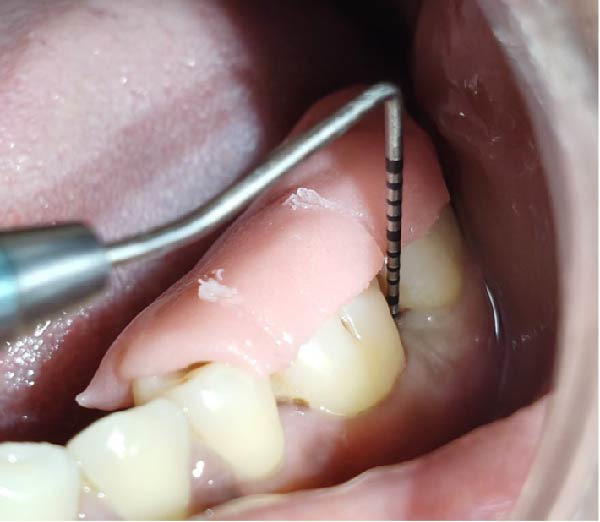
Measurement of PPD at baseline in test group.

**Figure 5:**
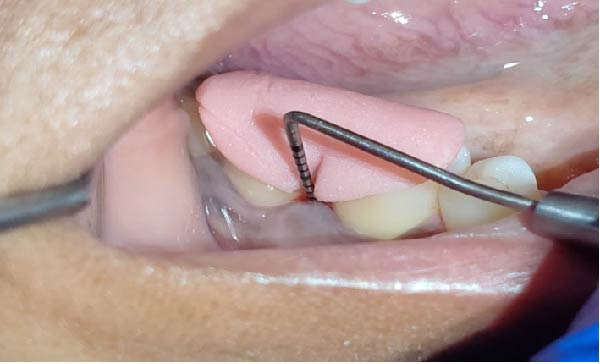
Measurement of PPD at baseline in control group.

**Figure 6:**
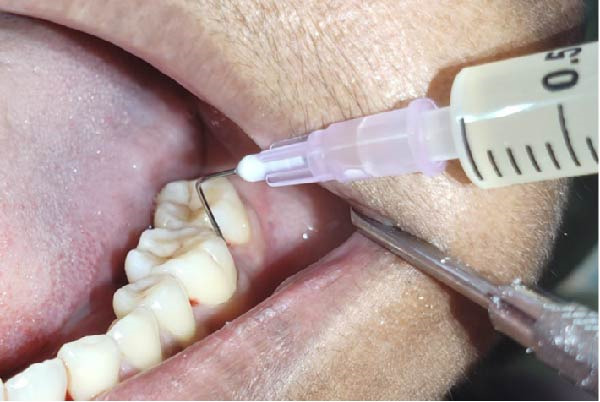
Application of CSPE gel into the pocket.

**Figure 7:**
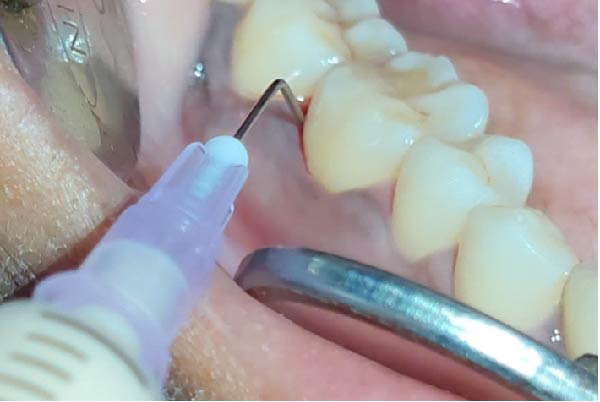
Application of CHX gel into the pocket.

Forty‐six subjects with Grade II/III localized periodontitis were randomly allocated into two groups. After thorough SRP, test group (*n* = 23) was treated with CSPE gel and control group (*n* = 23) with 1% CHX gel. Full mouth supragingival and sub gingival scaling was performed for both test (Figure 4) and control (Figure 5) group and patients were educated and motivated regarding plaque control. After full mouth preparation both the gels were applied in their respective allotted groups using 2 mL syringe. (Figures 6 and 7)

**Figure 8:**
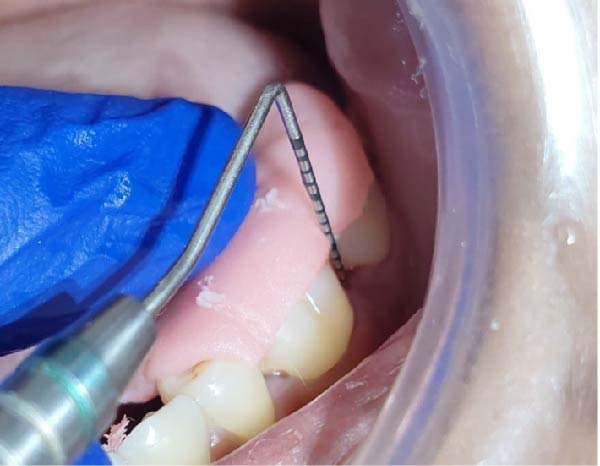
Reduction of PPD seen at 60 days in test group.

**Figure 9:**
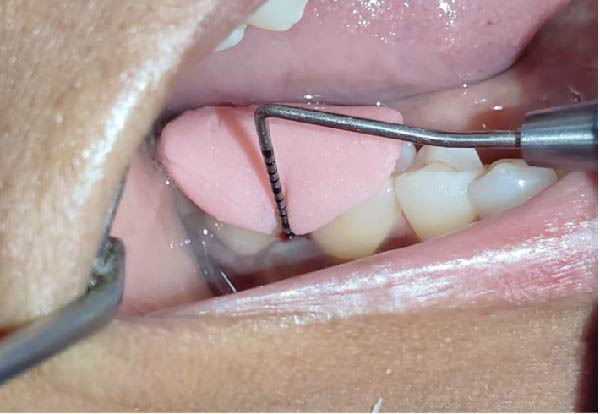
Reduction of PPD seen at 60 days in control group.

Clinical parameters measured were plaque index [PI (Silness and Loe, 1964)], PPD, clinical attachment level (CAL), at baseline (prior to SRP) and at 60 days (Figures 8 and 9) using University of North Carolina‐15 (UNC 15) periodontal probe [15]. A custom‐made acrylic occlusal stent was used for recording PPD and CAL.

### 2.4. Microbiological Analysis

Saliva contamination was controlled using sterile gauze. Sterile periodontal Gracey curette was used to collect subgingival plaque sample in peptone water in a test tube. It was transported to the microbiology laboratory and processed immediately. Total bacterial colony count or colony forming units (CFU) was determined from blood agar medium at baseline, prior to SRP and at 60 days post therapy for test group (Figure 10) and control group (Figure 11).Viable bacterial cells are measured in CFU and they represent individual colonies of particular organism.

Figure 10CFU at baseline (a) and at 60 days (b) for test group.

**Figure:**
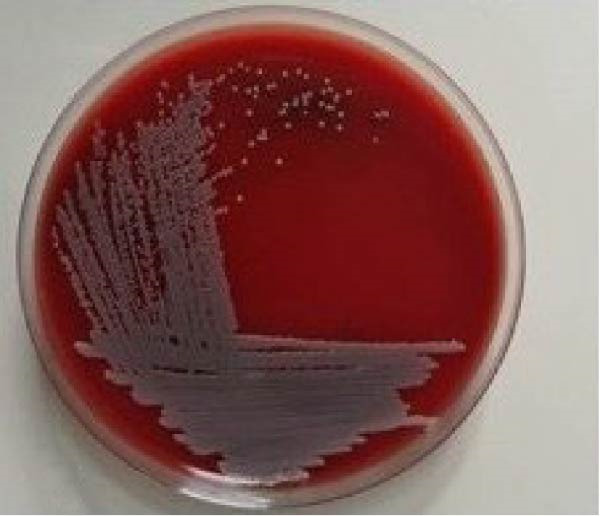
(a)

**Figure:**
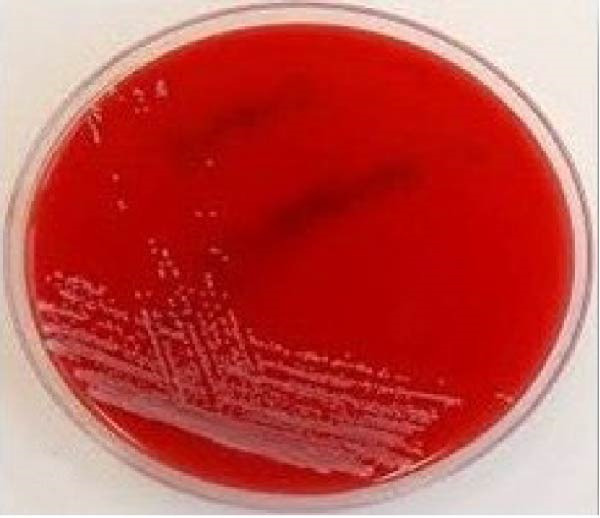
(b)

Figure 11CFU at baseline (a) and at 60 days (b) for control group.

**Figure:**
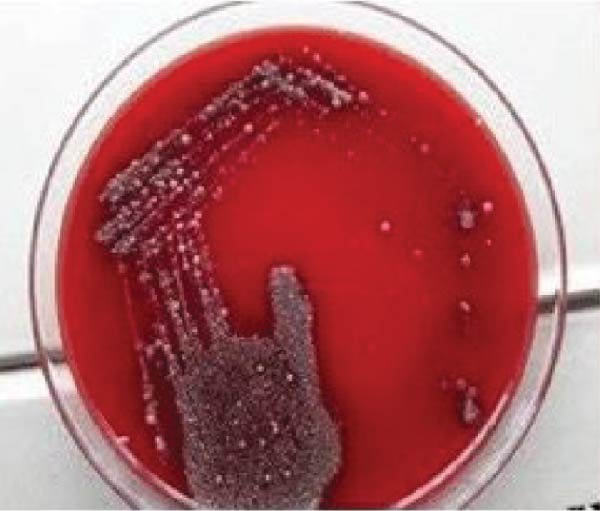
(a)

**Figure:**
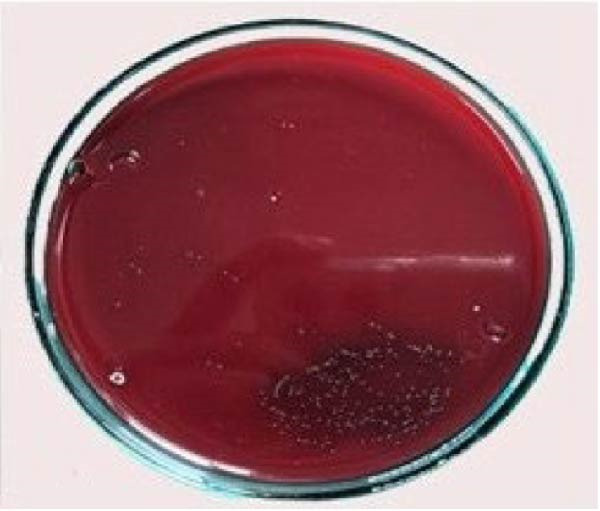
(b)

## 3. Results

**Figure 12:**
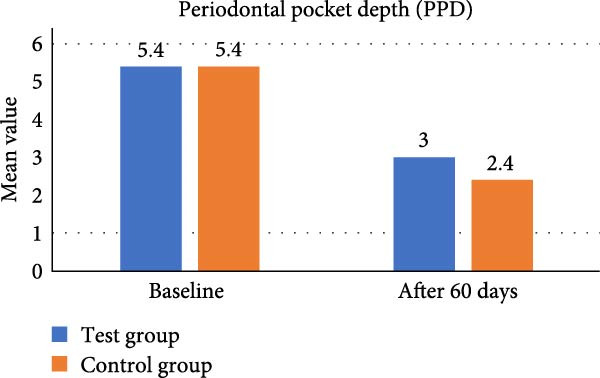
Comparative assessment of probing pocket depth at different time intervals among the study group.

In test group, mean value of PPD at baseline was 5.40 ± 0.51 and at 60 days reduced to 3.00 ± 0.66, resulted in improvement of PPD (*p* = 0.044). In control group, mean value of PPD at baseline was 5.40 ± 0.51 and at 60 days 2.40 ± 0.51, which showed *p* value 0.645. Post local drug delivery, at 60 days the PPD established between the test and control group (*p* = 0.037) (Table 1, Figure 12).

**Table 1:** Comparative assessment of periodontal parameters at different time intervals among the study groups.

Parameters	Baseline	After 60 days	*p*‐Value^@^
Periodontal pocket depth (PPD)			
Test group	5.40 ± 0.51	3.00 ± 0.66	0.044 ^∗^
Control group	5.40 ± 0.51	2.40 ± 0.51	0.645
* p*‐Value^#^	1.000	0.037 ^∗^	—
Clinical attachment level (CAL)			
Test group	4.40 ± 0.51	2.00 ± 0.66	0.044 ^∗^
Control group	4.40 ± 0.51	1.80 ± 0.78	0.356
* p*‐Value^#^	1.000	0.548	—
Bacterial colony count			
Test group	880.20 ± 45.13	554.20 ± 33.26	0.422
Control group	803.40 ± 42.76	470.80 ± 27.05	0.134
* p*‐Value^#^	0.001 ^∗^	0.001 ^∗^	—
Plaque index			
Test group	0.60 ± 0.51	0.80 ± 0.42	0.242
Control group	0.40 ± 0.51	0.60 ± 0.51	0.645
* p*‐Value^#^	0.398	0.355	—

*Note: p*‐Value–probability value, test applied.

^@^Paired *t* test.

^#^Independent *t* test.

^∗^Indicates statistically significant difference.

**Figure 13:**
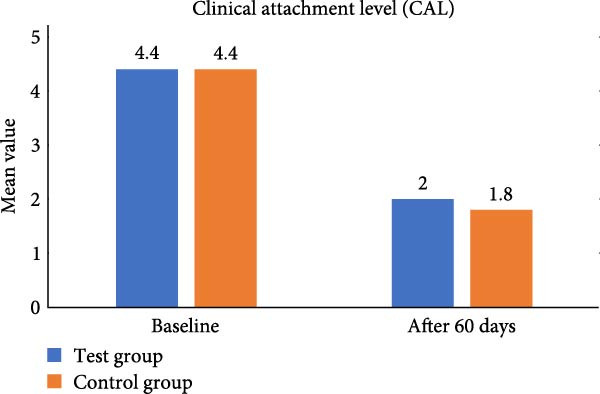
Comparative assessment of clinical attachment level at different time intervals among the study group.

In test group, mean value of CAL at baseline was 4.40 ± 0.51 which decreased to 2.00 ± 0.66 at 60 days and showed significant statistical difference (*p* = 0.044).In control group, mean value of CAL at baseline was 4.40 ± 0.51 and at 60 days was 1.80 ± 0.78 showed *p* value 0. 356. When we measured CAL between the two groups statistically, it was found nonsignificant (Table 1, Figure 13).

**Figure 14:**
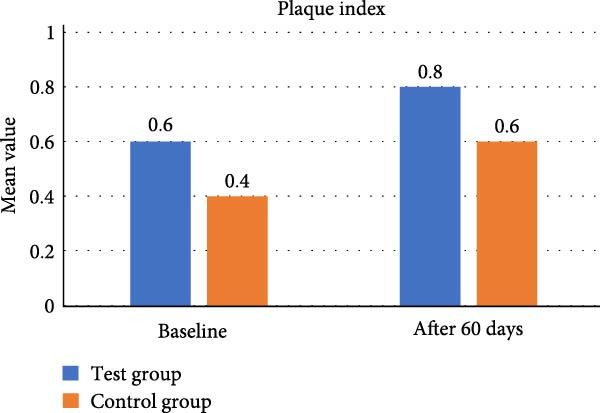
Comparative assessment of plaque index at different time intervals among the study group.

In test group, mean value of plaque index at baseline was 0.60 ± 0.51 and at 60 days it showed a reduction to 0.80 ± 0.42 with *p* = 0.242. In control group, mean value of plaque index at baseline was evaluated to 0.40 ± 0.51 and at 60 days 0.60 ± 0.51, which showed *p* value = 0.645. Inter group and intra group plaque index comparison showed no difference statistically (Table 1, Figure 14).

**Figure 15:**
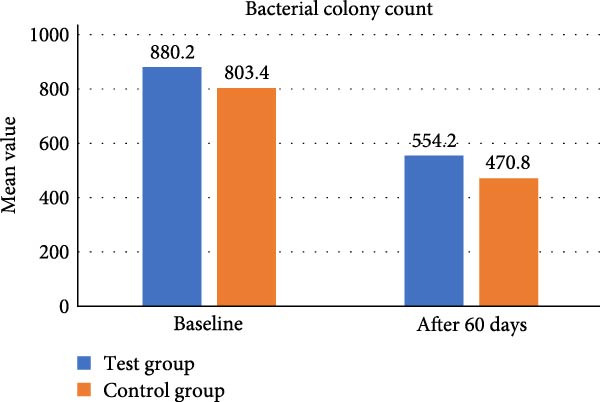
Comparative assessment of colony forming unit at different time intervals among the study group.

In test group, mean value of CFU at baseline was 880.20 ± 45.13 CFU which reduced to 554.20 ± 33.26 CFU at 60 days (*p* = 0.422).In control group, mean value of colony forming unit at baseline 803.40 ± 42.76 CFU and at 60 days it decreased to 470.80 ± 27.05 showed *p* value = 0.134. A statistically significant difference was found in colony forming unit between the two groups (*p* = 0.001) (Table 1, Figure 15).

## 4. Discussion

Phytotherapy has a vast scope in periodontal therapy. Natural extracts offer antioxidant, antimicrobial and minimal side effects. Mosaddad et al. [16] narrated the antibacterial properties of various natural products against periodontal pathogens in periodontal pockets oral microorganisms. So far, 0.2% or 0.12% CHX gluconate has been accounted as a touchstone for the possible prevention of gingival inflammation and further progression of periodontal diseases [17]. However, our study was designed to correlate the potency of orange peel gel and CHX gel as a supplement to SRP in localized periodontitis.

Pandey et al. [18] compared efficacy of curcumin gel along with Phase I therapy and found that the medicinal value of curcumin having high antioxidant capacity can be beneficial in reducing microbial count. The formulation of gel and initial dosage determination is critical in determining the efficacy of local drug delivery agents. Rao et al. [19] evaluated azithromycin 0.5% and doxycycline 5% as local drug delivery in periodontitis and found clinically effective in reducing *Porphyromonas gingivalis* count. Another study by Shashikumar et al. [20] evaluated use of *Morinda citrifolia* L. as antibacterial agent in form of phytotherapy agents in periodontal disease treatment. They found that *Morinda citrifolia* has high antioxidant value and is comparable with CHX.

Local drug delivery agents can be used in form of pellets for sustained release which can beneficial for long term controlled release of the drug. Nisha et al. [21] in their study on use on homeopathic medication *Hypericum perforatum* found similar efficacy of this medication as compared to CHX. Mouthwashes are chemical plaque control agents and they are used as over the counter medication as a home regimen to maintain oral hygiene. They are also used as preprocedural mouthrinses to reduce aerosol contamination [22–24. The consistency of local drug delivery agents varies as compared to mouthwashes.

Shashikumar et al. [25] used *Centella asiatica* in diabetes patient and found statistically significant difference compared to CHX. Plaque index, PPD was reduced in this study suggesting the potential role this phytotherapy agent in periodontal treatment. Hanafy et al. [26] performed an in‐vitro chemical profiling of orange peel and proved antimicrobial and antioxidant activities of citrus peel extract. In this study, we have assessed the clinico‐microbial outcome of orange peel extract gel/CSPE gel as LDD as an adjunct to SRP and is compared with CHX 1% gel in Grade II/III periodontitis patient. Clinical evaluation of our study showed CSPE LDD gel administered in periodontal pocket after SRP had significantly reduced pocket depth from mean PPD of 5.40 + 0.51 mm at baseline to 3.00 ± 0.66 mm at 60 days (*p* = 0.044). Simultaneously, CHX 1% LDD gel also showed mean PPD reduction from 5.40 ± 0.51 mm at baseline to 2.40 ± 0.51 mm at 60 days (*p* = 0.645). CSPE gel showed a statistically significant PPD reduction at 60 days compared to CHX gel LDD (*p* = 0.037).

Saha et al. [27] evaluated the antibiofilm efficacy of orange peel extract and found minimal inhibitory concentration against *Streptococcus mutans* as 13 + 2.0, based on this study, only our present study determined the drug dosage for local delivery for *Citrus sinensis*. Shetty et al. [13] determined the antimicrobial effect of orange peel extract against *Streptococcus mutans*. They found that this plant extract has high potential antimicrobial action against dental caries progression. However, no clinical study has evaluated efficacy against periodontal pathogen and our study is the first to report efficacy of *Citrus sinensis* as local drug delivery agent in the localized periodontitis treatment. Hussain et al. [28] in their in‐vitro study examined periodontal pathogens like *Prevotella intermedia* and *Porphyromonas gingivalis* resistant to aqueous extracts of *Citrus sinensis* and high efficacies against *Aggregatibacter actinomycetemcomitans*.

Both CSPE gel and CHX gel has also shown significant improvement and CAL gain, *p* = 0.044 and *p* = 0.356 respectively. CSPE gel shows however comparatively better outcome in gain of clinical attachment. Clinical evaluation of our study showed CSPE LDD gel application in periodontal pocket after SRP had significant improvement in attachment level from mean CAL of 4.40 ± 0.51 at baseline to 2.00 ± 0.66 at 60 days (*p* = 0.044). Simultaneously, CHX 1% LDD gel also showed mean CAL reduction from 4.40 ± 0.51 at baseline to 1.80 ± 0.78 at 60 days (*p* = 0.356). CSPE gel showed comparable outcome in clinical attachment gain with CHX 1% gel. In another study Maruti et al. [29] showed 44 % of the Gram +ve and 69% of the Gram ‐ve pathogens showed the 10 mm diameter of inhibition zone citrus peel extract. In the present study on evaluation in blood agar plate, total colony forming unit at baseline was 880.20 ± 45.13 CFU which reduced to 554.20 ± 33.26 CFU at 60 days (*p* = 0.422) using CSPE gel. In control group, Colony Forming Unit at baseline 803.40 ± 42.76 CFU and at 60 days it decreased to 470.80 ± 27.05 (*p* = 0.134). Colony forming unit between the two groups was found statistically significant (*p* = 0.001).

Boyapati et al. [30] investigated the efficacy of *Achyranthes aspera* gel in chronic periodontitis and found that this herbal agent has antimicrobial and immunomodulatory activity contributing in reduction towards periodontal inflammation. We also propose that *Citrus sinensis* peel extract (CSPE) gel has an antioxidant and immunomodulatory activity which might be responsible for reduce PPD. Though we didn’t measure the antioxidant activity but this will be future recommendation to measure total antioxidant activity of this extract gel which can further help us in standardization of the gel dosage. Herbal formulations have been used in periodontal disease treatment but lack of proper drug dosage restricts the replication of study [31]. This can be considered as a limitation of the present study also. Generalization of gel efficacy and dosage cannot be done and large sample multicenter study can be considered to overcome this challenge. Further, we would propose to determine different dosage of orange peel extract and other herbal drugs in the treatment of both nonsurgical and surgical periodontal therapy.

In our present study, according to the microbiological analysis, mean CFU was reduced to 554.20 ± 33.26 at 60 days from 880.20 ± 45.13 at baseline using CSPE LDD (*p* = 0.422). Similarly using 1% CHX LDD showed 470.80 ± 27.05 CFU at 60 days from 803.40 ± 42.76 CFU at baseline (*p* = 0.134). CFU between the two groups (*p* = 0.001) was found statistically significant. In test group, mean value of colony forming unit at baseline was 880.20 ± 45.13 CFU which reduced to 554.20 ± 33.26 CFU at 60 days (*p* = 0.422).In control group, mean value of CFU at baseline 803.40 ± 42.76 CFU and at 60 days it decreased to 470.80 ± 27.05 showed *p* value = 0.134. A statistically significant difference was found in CFU between the two groups (*p* = 0.001). Plaque was well maintained in both the groups throughout the study period as evident by PI score. Thus, this present study showed CSPE Gel LDD provides a comparable outcome with conventional CHX 1% gel in terms of antibacterial effect on periodontal pocket.

## 5. Conclusions

The current study revealed that CSPE can be used as local drug delivery agent against periodontal pathogens. Different concentrations of extract can be prepared which would determine the correct dosage of extract and its efficacy. In‐vitro and animal studies can help in dosage determination. Phytotherapy is a boon to periodontal therapy and acts as potential immunomodulator reducing the oxidative stress and uplifting the patient’s immune response.

## Conflicts of Interest

The authors declare no conflicts of interest.

## Author Contributions

Ishika Sarkar, Swet Nisha, Pritish Chandra Pal, S. R. Savan, Debika Karmakar, and Dhanraj Budhai contributed equally to this work.

## Funding

This research received no grant from any funding agency.

## Data Availability

The data that support the findings of this study are available from the corresponding author upon reasonable request.
